# Effects of metals released in strong‐flavor baijiu on the evolution of aroma compounds during storage

**DOI:** 10.1002/fsn3.1475

**Published:** 2020-02-20

**Authors:** Zhangjun Huang, Yunhang Zeng, Wenhu Liu, Songtao Wang, Caihong Shen, Bi Shi

**Affiliations:** ^1^ College of Biomass Science and Engineering Sichuan University Chengdu China; ^2^ National Engineering Research Center of Solid‐State Brewing Luzhou China; ^3^ Luzhou Laojiao Co. Ltd Luzhou China

**Keywords:** aroma compounds, metals, storage, strong‐flavor baijiu

## Abstract

Storage is essential in improving the quality of strong‐flavor baijiu (SFB). Here, we investigated the release behaviors of metals from containers into SFB and their effects on the evolution of aroma compounds during storage. Twenty‐six metals were identified in SFB samples. The concentrations of Na, K, Ca, Mg, Al, and Fe obviously increased after storing in pottery jar, whereas those of Fe and Cu greatly increased after storing in stainless‐steel vessel. The volatility of most esters, alcohols, ketone, furan, and aldehyde decreased, whereas that of most acids increased after adding the metal ions into fresh SFB. The fluorescence intensity of SFB decreased with increased aging time in pottery jar, whereas the fluorescence intensity of acids was quenched with adding Fe^3+^ and Cu^2+^. All these results suggested that some metals released from containers had binding affinities with acids, thereby reducing SFB organoleptic stimulation by forming metal‐aroma compound complexes during storage.

## Graphical abstract

Some metals were dissolved out from containers such as pottery jar and stainless‐steel vessel into strong‐flavor baijiu (SFB) during storage. These metals had binding affinities with aroma compounds, thereby reducing SFB organoleptic stimulation by forming metal‐aroma compound complexes during storage.

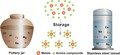

## 1 INTRODUCTION

Baijiu is an ancient Chinese liquor that belongs the six major distilled spirits in the world. Baijiu has a distinctive flavor different from other major distilled spirits, such as brandy, whiskey, rum, gin, and vodka (Jin, Zhu, & Xu, 2017; Liu & Sun, 2018). The flavors of baijiu are generally classified into twelve types, including three major and nine minor flavor types (Zheng & Han, 2016). Strong‐flavor baijiu (SFB) is one of the major flavor types and accounts for approximately 70% of the total baijiu production in China. Production of SFB is unique and complex, involving solid‐state fermentation, distillation, storage/aging, and blending. Sorghum or a mixture of corn, rice, glutinous rice, and wheat are mostly used as raw materials for SFB production, and daqu is used as the saccharifying and fermentative agent. Fermentation is carried out in a mud pit (Xu et al., 2017; Zou, Zhao, & Luo, 2018). As a result, SFB is rich in volatile compounds, including esters, acids, alcohols, aldehydes, ketones, pyrazines, furans, phenols, aromatics, and sulfides (Liu & Sun, 2018; Wang, Fan, & Xu, 2014). Furthermore, SFB contains some nonvolatile compounds, such as metals, amino acids, and peptides, which have potential functions and are beneficial to human health (Cui, 2008; Song et al., 2018; Zhang, Wu, Xu, & Qian, 2014).

Storage is an essential procedure in SFB production. Fresh distilled baijiu with unpleasant, rough and pungent flavor needs to be stored in a pottery jar or a stainless‐steel vessel for months or years to improve SFB quality (Zheng & Han, 2016). After storage, fresh distilled baijiu is converted into aged/stored SFB, and its flavor becomes mellow and harmonious, and even appears chen‐aroma. During storage, some metals are released into SFB from the containers (Jiang, Xie, Wan, Chen, & Zheng, 2019). These metals can affect the taste and smell of baijiu. Xiong, Xiang, and Zhao (2013) reported that Ca, Fe, Mg, and Cu released from pottery jar into baijiu improved the soft mellow and harmonious mouthfeel. However, the relationship between metals and flavor compounds of SFB, which is closely related to SFB quality, remains unclear because the flavor compounds are too complex.

Metals play an important role in modulating sensory attributes of alcoholic beverage. Cu, Fe, Mn, Al, and Zn influence the release and formation of some aroma compounds, such as hydrogen sulfide, methanethiol, and dimethyl sulfide in wine (Franco‐Luesma & Ferreira, 2014; Viviers, Smith, Wilkes, & Smith, 2013). The presence of Cu at a low level leads to a better taste and aroma of brandy (Bonic et al., 2013). Therefore, understanding the effects of metals on the release and formation of aroma compounds of baijiu are important for modulating its flavor and sensory properties.

This work aimed to investigate the effects of metals released from pottery jar or stainless‐steel vessel on the evolution of aroma compounds of SFB during storage. Release behaviors of metals into SFB were first investigated. Major metals Na, K, Ca, Mg, Al, and Fe in SFB samples were determined via inductively coupled plasma‐optical emission spectrometry (ICP‐OES), and trace metals Ag, As, Ba, Be, Cd, Cr, Cu, Li, Mn, Ni, Pb, Sr, Ti, Zn, La, Pr, Nd, Sm, Eu, and Gd were determined via inductively coupled plasma‐mass spectrometry (ICP‐MS). Then, changes of aroma compounds in SFB after adding various metal ions were analyzed via headspace solid‐phase microextraction combined with gas chromatography–mass spectrometry (HS‐SPME‐GC‐MS), and the metal‐binding abilities of aroma compounds were evaluated via fluorescence spectroscopy to further understand the effects of metals on aroma compounds of SFB.

## 2 MATERIALS AND METHODS

### 2.1 Materials

SFB samples were the same level base baijiu and provided by Luzhoulaojiao Co., Ltd. (Luzhou, China). Samples were stored in pottery jar or type 304 stainless‐steel vessel for 0, 1, 2, 3, 5, and 10 years. The volume of the pottery jar is 1 m^3^, and its surface area is approximately 5.8 m^2^ in comparison to 1 m^3^ of SFB. The volume of the stainless‐steel vessel is 10 m^3^, and its surface area is approximately 2.1 m^2^ in comparison to 1 m^3^ of SFB. During storage, the pottery jar was sealed and put in a cave at 20°C. The stainless‐steel vessel was sealed and put in the shade at a room temperature which varied with the seasons. Standard solutions of Na, K, Ca, Mg, Al, Fe, Ag, As, Ba, Be, Cd, Cr, Cu, Li, Mn, Ni, Pb, Sr, Ti, Zn, La, Pr, Nd, Sm, Eu, and Gd were purchased from Guobiao Testing & Certification Co., Ltd. Acetic acid, butanoic acid, and hexanoic acid (GC grade, ≥98% purity) were purchased from Dr. Ehrenstorfer. Internal standard, methyl hexanoate, was purchased from ANPLE Scientific Instrument Co., Ltd. A C_7_–C_40_ n‐alkane mixture (O2SI Smart Solutions) was employed to determine retention indices (RIs). Ethanol (high‐performance liquid chromatography grade, 99% purity), nitric acid, hydrogen peroxide, NaCl, KCl, CaCl_2_, MgCl_2_•6H_2_O, AlCl_3_•6H_2_O, FeCl_3_•6H_2_O, and CuCl_2_•2H_2_O were of analytical grade and purchased from Kelong Chemical Co., Ltd. Milli‐Q water (18.2 MΩ/cm resistivity) was prepared through a Milli‐Q system (Millipore).

### 2.2 Analysis of metals in the stored SFB by ICP‐OES and ICP‐MS

SFB samples (25 ml) were heated at 80°C until their volume was reduced to 2–3 ml. Then, the concentrated samples were digested at 120°C with 5 ml nitric acid and 1 ml hydrogen peroxide. After cooling to room temperature, these digested solutions were diluted to 25 ml with Milli‐Q water. The concentrations of major metals, such as Na, K, Ca, Mg, Al, and Fe, were determined by ICP‐OES (Optima 8000; PerkinElmer), and the concentrations of trace metals, such as Ag, As, Ba, Be, Cd, Cr, Cu, Li, Mn, Ni, Pb, Sr, Ti, Zn, La, Pr, Nd, Sm, Eu, and Gd, were determined by ICP‐MS (ELAN 9000/DRC‐e; PerkinElmer).

### 2.3 HS‐SPME extraction and GC‐MS analysis of aroma compounds in SFB

Aqueous solutions of NaCl, KCl, CaCl_2_, MgCl_2_, AlCl_3_, FeCl_3_, and CuCl_2_, in which the metal ion has a concentration of 1,000 mg/L, were prepared individually. Different volumes of the above‐mentioned solutions were added into fresh SFB samples to obtain 2 and 10 mg/L of Na^+^, K^+^, and Ca^2+^, 0.5 and 2 mg/L Mg^2+^, Al^3+^, Fe^3+^, and Cu^2+^. SFB samples with addition of metal were regarded as experimental groups, and SFB sample without addition of metal was the control group. All samples were stored at 25°C for a week.

Changes in aroma compounds of fresh SFB after adding various metal ions were analyzed through HS‐SPME‐GC‐MS using the reported method with some modifications (Huang et al., 2019; Zhang, Wu, & Xu, 2014). SFB sample (1 ml) and methyl hexanoate (10 μL, 1,310 μg/L in 70% ethanol/water solution v/v) as internal standard were added into a 20‐mL screw‐capped headspace vial. The vial was placed in thermostatic water bath, equilibrated at 40°C for 10 min and extracted for 45 min at the same temperature by being exposed to a 50/30 μm divinylbenzene/carboxen/polydimethylsiloxane fiber (2 cm; Supelco, Inc.). Subsequently, the fiber was transferred into an injector to desorb at 250°C for 5 min.

Volatile aroma compounds were then analyzed via GC‐MS (QP2020; Shimazu, Japan). HP‐Innowax column (60 m × 0.25 mm i.d. × 0.25 μm; Agilent) was used as capillary column. Helium (purity 99.999%) was used as carrier at a constant flow rate of 1 ml/min. Injector was maintained at 250°C. Oven temperature was started at 40°C for 5 min, raised to 100°C at rate of 4°C/min, then ramped to 230°C at a rate of 6°C/min and held for 15 min. For MS analysis, electron ionization was used at 70 eV. Temperatures of the interface and the ion source were set at 250 and 230°C, respectively. The mass range scanned was from 30 to 450 amu at a scan rate of 0.3 s.

Volatile aroma compounds were identified by comparing their mass spectra (MS) with the NIST14 mass spectra database and comparing their retention indices (RIs) with those of reference compounds. Net changes were used to evaluate aroma volatility and were calculated according to the equation: Net change (%) = (mean peak area of control—mean peak area of experimental group)/ mean peak area of control × 100% (Huang et al., 2018; Zhang et al., 2014).

### 2.4 Fluorescence spectra of SFB and acids solution

Fluorescence excitation‐emission matrix (EEM) spectra of the SFB samples stored in pottery jar for 0, 1, 2, 3, 5, and 10 years were measured using a fluorescence spectrometer (F‐7000; Hitachi). Excitation and emission wavelengths were recorded from 200 nm to 350 nm and 200 nm to 450 nm, respectively, with 2.5 nm slit widths.

Different volumes of NaCl, KCl, CaCl_2_, MgCl_2_, AlCl_3_, FeCl_3_, and CuCl_2_ aqueous solutions, in which metal ion concentration was 2000 mg/L, were added into fresh SFB samples to obtain different concentrations of Na^+^ (0.5, 5, 20, and 100 mg/L), K^+^ (0.5, 5, 20, and 100 mg/L), Ca^2+^ (0.5, 5, 20, and 100 mg/L), Mg^2+^ (0.5, 5, 20, and 100 mg/L), Al^3+^ (0.5, 2, 10, and 50 mg/L), Fe^3+^ (0.5, 2, 10, and 50 mg/L), and Cu^2+^ (0.2, 0.5, 2, and 10 mg/L). Fresh SFB samples with metal ions were stilled for a week. Then, fluorescence intensities at the maximum emission wavelength of the samples were recorded with the maximum excitation wavelength of 280 nm. F_0_ and F were the fluorescence intensities of the SFB with and without metal ions, respectively.

Fluorescence emission spectra of acid solutions with different concentrations of a metal ion (Na^+^, K^+^, Ca^2+^, Mg^2+^, Al^3+^, Fe^3+^, or Cu^2+^) were recorded at maximum excitation wavelength of 220 nm. Acid solutions include 0.5 g/L of acetic acid, butanoic acid, and hexanoic acid in 70% (v/v) ethanol/water. Metal ion concentrations were 0, 0.1, 0.5, 2, 5, 10, 20, and 50 mg/L.

### 2.5 Statistical analysis

Each sample was analyzed in triplicate for each determination. The results in tables are presented as mean ± standard deviation. One‐way analysis of variance (ANOVA) and Duncan's multiple range tests were carried out with SPSS 25.0 software to evaluate significant differences of assay (*p* < .05).

## 3 RESULTS AND DISCUSSIONS

### 3.1 Quantification of metals in SFB

Twenty‐six metals in fresh and stored SFB samples were analyzed via ICP‐OES and ICP‐MS, and the results are listed in Table 1. Na, K, Ca, Mg, Al, and Fe are the major metals with high content in baijiu (Xie, Jiang, & Wu, 2017). Hence, their concentrations were determined by ICP‐OES, which is usually used to analyze samples with high total dissolved solids content. Besides, the minor metals Ag, As, Ba, Be, Cd, Cr, Cu, Li, Mn, Ni, Pb, Sr, Ti, Zn, La, Pr, Nd, Sm, Eu, and Gd were analyzed by ICP‐MS, which is more sensitive, but limited to samples with low total dissolved solids content (Martin, Watling, & Lee, 2012; Šelih, Šala, & Drgan, 2014). Fresh SFB had little amounts of metals because most of metals remained in Zaopei after distillation (Li et al., 2016; Zou et al., 2018). However, some metals were dissolved out from containers during storage. SFB sample stored in pottery jar for 10 years contained 4,245.333 μg/L Ca, 1643.333 μg/L Na, 1548.000 μg/L K, 1,028.667 μg/L Al, 390.333 μg/L Mg, and 328.333 μg/L Fe. These metal concentrations were remarkably higher than those in other samples. The maximum concentration of Cu (336.667 μg/L) was achieved in SFB stored in stainless‐steel vessel for 5 years. These metal concentrations found in stored samples were below the international statutory limits of alcoholic beverages (Iwegbue, Overah, Bassey, & Martincigh, 2014). Concentrations of Ba, Cr, Mn, Ni, Sr, Ti, and Zn were in the range of 0.093–46.959 μg/L, and Li concentration ranged from 0.003 to 14.901 μg/L. Concentrations of trace metals Ag, As, Be, Cd, and Pb were <1.289 μg/L, and concentrations of La, Pr, Nd, Sm, Eu, and Gd were below 0.5 μg/L in all samples. Relatively low levels of major and trace metals detected in stored SFB indicated that metal concentrations released from pottery jar and stainless‐steel vessel during storage did not exceed the recommended allowable daily intake according to the alcohol control database of the WHO (Bonic et al., 2013; Iwegbue, Overah, Bassey, & Martincigh, 2014; Salako, Adekoyeni, Adegbite, & Hammed, 2016).

**Table 1:** Metal concentrations (μg/L) in SFB stored in pottery jar and stainless‐steel vessel

Metal	P0/S0	P1	P3	P5	P10	S1	S3	S5	S10
Na	76.633 ± 6.435^d^	74.467 ± 4.100^d^	184.667 ± 16.503^c^	348.000 ± 23.516^b^	1643.333 ± 19.296^a^	57.800 ± 4.214^d^	66.833 ± 7.112^d^	63.767 ± 3.383^d^	63.433 ± 4.875^d^
K	25.500 ± 2.879^d^	44.067 ± 3.729^d^	262.667 ± 11.504^c^	553.333 ± 37.754^b^	1548.000 ± 58.275^a^	25.267 ± 2.815^d^	31.133 ± 2.627^d^	52.733 ± 5.395^d^	53.467 ± 2.695^d^
Ca	60.900 ± 1.217^e^	137.000 ± 8.888^d^	822.000 ± 29.309^c^	1,447.667 ± 21.127^a^	4,245.333 ± 96.106^a^	61.933 ± 7.228^e^	103.333 ± 8.505^de^	126.333 ± 13.051^de^	158.000 ± 26.211^d^
Mg	8.783 ± 0.958^e^	20.933 ± 2.676^de^	137.000 ± 11.000^c^	235.667 ± 5.033^b^	390.333 ± 32.005^a^	7.627 ± 0.862^e^	34.300 ± 0.458^d^	33.967 ± 2.589^d^	42.000 ± 3.606^d^
Al	21.733 ± 2.663^e^	32.267 ± 1.804^e^	343.000 ± 13.077^c^	567.333 ± 69.644^b^	1,028.667 ± 32.347^a^	24.333 ± 1.060^e^	33.900 ± 3.439^e^	114.333 ± 10.970^d^	148.667 ± 19.655^d^
Fe	9.473 ± 1.119^f^	15.133 ± 0.586^f^	112.133 ± 9.201^e^	216.667 ± 26.690^c^	328.333 ± 20.306^a^	25.700 ± 3.020^f^	87.807 ± 3.694^e^	140.333 ± 23.544^d^	268.667 ± 10.504^b^
Ag	0.015 ± 0.004^de^	0.010 ± 0.002^ef^	0.033 ± 0.004^b^	0.045 ± 0.003^a^	0.021 ± 0.004^d^	0.018 ± 0.003^d^	0.015 ± 0.002^de^	0.027 ± 0.006^c^	0.007 ± 0.001^f^
As	0.160 ± 0.017^d^	0.210 ± 0.001^d^	0.414 ± 0.031^c^	0.745 ± 0.095^b^	1.289 ± 0.256^a^	0.141 ± 0.005^d^	0.175 ± 0.007^d^	0.776 ± 0.135^b^	0.874 ± 0.053^b^
Ba	0.093 ± 0.013^c^	0.140 ± 0.016^c^	0.267 ± 0.020^c^	0.749 ± 0.159^b^	2.700 ± 0.269^a^	0.181 ± 0.022^c^	0.155 ± 0.030^c^	0.234 ± 0.020^c^	0.260 ± 0.046^c^
Be	0.001 ± 0.000^e^	0.001 ± 0.000^e^	0.017 ± 0.002^c^	0.080 ± 0.003^b^	0.107 ± 0.004^a^	0.001 ± 0.000^e^	0.001 ± 0.000^e^	0.011 ± 0.002^d^	0.016 ± 0.003^c^
Cd	0.003 ± 0.001^c^	0.002 ± 0.001^c^	0.008 ± 0.002^bc^	0.044 ± 0.006^a^	0.037 ± 0.015^a^	0.013 ± 0.002^bc^	0.040 ± 0.010^a^	0.016 ± 0.004^b^	0.041 ± 0.002^a^
Cr	0.331 ± 0.004^d^	0.436 ± 0.039^d^	1.828 ± 0.081^c^	2.254 ± 0.890^c^	1.783 ± 0.228^c^	1.310 ± 0.131^cd^	2.362 ± 0.104^c^	4.167 ± 1.102^b^	12.229 ± 1.154^a^
Cu	0.110 ± 0.015^c^	0.092 ± 0.012^c^	2.152 ± 0.149^c^	4.818 ± 0.428^c^	8.303 ± 0.536^c^	0.309 ± 0.054^c^	1.138 ± 0.132^c^	336.667 ± 21.733^a^	196.667 ± 27.301^b^
Li	0.003 ± 0.001^e^	0.205 ± 0.015^e^	2.267 ± 0.201^c^	6.989 ± 0.058^b^	14.901 ± 1.152^a^	0.022 ± 0.004^e^	0.493 ± 0.069^de^	1.185 ± 0.029^d^	0.515 ± 0.058^de^
Mn	0.555 ± 0.031^f^	0.732 ± 0.031^f^	3.877 ± 0.298^e^	9.097 ± 0.199^d^	15.861 ± 1.214^c^	0.731 ± 0.012^f^	1.687 ± 0.055^f^	19.329 ± 1.533^b^	29.306 ± 1.563^a^
Ni	1.049 ± 0.070^e^	0.754 ± 0.038^e^	1.656 ± 0.126^e^	6.010 ± 0.074^b^	2.976 ± 0.197^d^	1.693 ± 0.189^e^	1.127 ± 0.044^e^	4.140 ± 0.803^c^	10.441 ± 1.258^a^
Pb	0.053 ± 0.008^f^	0.073 ± 0.010^ef^	0.164 ± 0.007^de^	0.309 ± 0.007^c^	0.614 ± 0.095^b^	0.221 ± 0.046^cd^	0.152 ± 0.037^def^	0.842 ± 0.118^a^	0.746 ± 0.073^a^
Sr	0.232 ± 0.014^e^	0.278 ± 0.038^e^	5.121 ± 0.376^c^	7.803 ± 0.189^b^	15.247 ± 0.427^a^	0.205 ± 0.013^e^	0.293 ± 0.022^e^	1.866 ± 0.225^d^	2.003 ± 0.091^d^
Ti	1.123 ± 0.123^e^	2.657 ± 0.581^de^	6.482 ± 0.498^b^	5.417 ± 0.584^bc^	20.651 ± 3.536^a^	1.136 ± 0.228^e^	2.626 ± 0.198^de^	3.757 ± 0.532^cd^	7.639 ± 0.103^b^
Zn	1.033 ± 0.040^e^	0.921 ± 0.116^e^	10.644 ± 0.684^d^	13.137 ± 1.051^d^	26.269 ± 3.161^c^	1.295 ± 0.176^e^	3.609 ± 0.119^e^	46.959 ± 2.893^a^	34.345 ± 7.079^b^
La	0.062 ± 0.006^e^	0.104 ± 0.009^d^	0.119 ± 0.005^d^	0.165 ± 0.022^c^	0.251 ± 0.012^b^	0.046 ± 0.002^e^	0.057 ± 0.003^e^	0.047 ± 0.006^e^	0.454 ± 0.054^a^
Pr	0.012 ± 0.002^e^	0.024 ± 0.001^d^	0.029 ± 0.002^d^	0.043 ± 0.005^c^	0.074 ± 0.007^b^	0.010 ± 0.001^e^	0.012 ± 0.002^e^	0.012 ± 0.001^e^	0.109 ± 0.003^a^
Nd	0.045 ± 0.004^f^	0.091 ± 0.012^e^	0.116 ± 0.002^d^	0.174 ± 0.018^c^	0.276 ± 0.009^b^	0.033 ± 0.004^f^	0.042 ± 0.009^f^	0.043 ± 0.001^f^	0.422 ± 0.009^a^
Sm	0.009 ± 0.001^ef^	0.019 ± 0.002^d^	0.028 ± 0.003c	0.035 ± 0.005^b^	0.065 ± 0.005^a^	0.006 ± 0.001^f^	0.012 ± 0.002^e^	0.004 ± 0.001^f^	0.009 ± 0.001^ef^
Eu	0.005 ± 0.001^c^	0.008 ± 0.001^b^	0.007 ± 0.001^bc^	0.008 ± 0.001^b^	0.019 ± 0.002^a^	0.006 ± 0.001^bc^	0.007 ± 0.002^b^	0.007 ± 0.001^b^	0.001 ± 0.000^d^
Gd	0.032 ± 0.003^e^	0.058 ± 0.005^bc^	0.049 ± 0.003^cd^	0.071 ± 0.011^b^	0.123 ± 0.018^a^	0.034 ± 0.005^de^	0.039 ± 0.003^de^	0.061 ± 0.006^bc^	0.125 ± 0.010^a^

Note

Values are mean ± standard deviation of triplicates. P0, P1, P3, P5, and P10 are SFB samples stored in pottery jar for 0, 1, 3, 5, and 10 years, respectively. S0, S1, S3, S5, and S10 are SFB samples stored in stainless‐steel vessel for 0, 1, 3, 5, and 10 years, respectively.

Values in the same row with different superscript letters are significantly different (*p* < .05).

The concentrations of most metals in SFB increased during storage (Figure S1). When SFB was stored in pottery jar, the concentrations of Na, K, Ca, Mg, Al, and Fe increased considerably, and concentrations of Ba, Be, Cu, Li, Mn, Ni, Sr, Ti, and Zn increased slightly. Pottery jar is made from fired clay and covered with glaze over the surface. Its main components are SiO_2_ and Al_2_O_3_ and contain small amounts of metallic oxides, such as CaO, Fe_2_O_3_, CuO, NiO, and TiO_2_ (Schleicher, Miller, Watkins‐Kenney, Carnes‐McNaughton, & Wilde‐Ramsing, 2008). These metallic oxides can be slowly dissolved into baijiu during storage (Yang, Zhao, & Liu, 2001); therefore, most metals in SFB increased during storage in pottery jar. When SFB was stored in stainless‐steel vessel, concentrations of Fe and Cu in SFB increased greatly and concentrations of Cr, Mn, Ni, Sr, Ti, and Zn increased slightly. The Cu in SFB should be the impurity, which was introduced from welding process in production of the stainless‐steel vessel (Magnabosco, Ferro, Bonollo, & Arnberg, 2006).

Metals in alcoholic beverages have a two‐faced role. Metals of proper amount and type are beneficial to modulating the flavor of alcoholic beverages and human health. For example, Fe and Cu can remove bad odors, and K, Ca, Cu, Zn, Fe, and Mg are essential metals in the human body (Ibanez, Carreon‐Alvarez, Barcena‐Soto, & Casillas, 2008). However, excess metals in alcoholic beverages would negatively influence their organoleptic quality and flavor and cause harm to human health. Excess Cu can give unpleasant tastes, and excess Fe changed the color of spirits to yellow or even brown (Pohl, 2007). Hence, metal concentrations in SFB should be limited according to the international statutory limits in alcoholic beverages recommended by the WHO.

### 3.2 Effect of main metals on aroma compounds in SFB

Na, K, Ca, Mg, Al, Fe, and Cu, which had relatively high concentrations in stored SFB, were chosen for further study of their effects on volatile aroma compounds. HS‐SPME method is widely used for identification and quantification of volatile components in baijiu (Fan & Qian, 2005; Zhang et al., 2014). In this study, a total of 30 volatile aroma compounds, including 17 esters, 6 alcohols, 4 acids, 1 ketone, 1 aldehyde, and 1 furan, were identified in SFB and quantified through the HS‐SPME method (Table 2). Addition of 2 mg/L Na^+^, K^+^, Ca^2+^, Mg^2+^, Al^3+^, Fe^3+^, or Cu^2+^ to the fresh SFB resulted in different effects on the headspace concentrations of esters, alcohols, acids, ketone, furan, and aldehyde. For esters, the volatility in the experimental groups largely decreased compared with the control group. When Na^+^ was added, the volatility of esters decreased by 0.04%–15.84%, except ethyl pentanoate and propyl hexanoate, which increased by 0.07% and 0.38%, respectively. After adding K^+^, the volatility of some esters decreased by 0.22%–20.22% and others increased by 1.08%–7.93%. Addition of Ca^2+^, Mg^2+^ or Fe^3+^ decreased the volatility of esters by 3.28%–33.41%, 1.71%–37.63%, and 0.58%–31.83%, respectively. When Al^3+^ was added, the volatility of most esters decreased by 1.34%–13.22% except ethyl lactate, pentyl hexanoate, and hexyl hexanoate. After adding Cu^2+^, the volatility of some esters decreased by 1.21%–18.89%, and others increased by 0.88%–9.14%. For alcohols, volatility decreased by 0.50%–41.65% in the experimental groups, except that the headspace concentration of 1‐propanol increased by 4.20% after adding Fe^3+^. For acids, the volatility of most acids in the experimental groups increased, except that the volatility of acetic acid, butanoic acid, and pentanoic acid decreased by addition of K^+^ and Fe^3+^. For ketone, furan, and aldehyde, the headspace concentrations decreased by 0.50%–24.90% after adding metals. However, concentrations of 2‐nonanone increased by 0.98% after adding K^+^; concentrations of 2‐nonanone and furfural increased by 3.62% and 1.59%, respectively, after adding Al^3+^; and furfural concentration increased by 6.12% after adding Cu^2+^.

**Table 2:** Effect of metal ions on mean HS‐SPME‐GC‐MS peak areas for aroma volatility in SFB

Compounds	RI	Odor	Identification	Control (μg/L)	Net change (%)						
					Na^+^	K^+^	Ca^2+^	Mg^2+^	Al^3+^	Fe^3+^	Cu^2+^
Ethyl acetate	889	Pineapple	MS, RI	160,722.8 ± 10,996.7	2.11	5.31	3.95	11.20	10.52	4.00	14.83
Ethyl butanoate	1,040	Pineapple	MS, RI	96,505.2 ± 1,216.3	2.39	6.11	8.03	8.16	7.85	3.45	1.57
Ethyl pentanoate	1,135	Apple	MS, RI	17,082.5 ± 1,190.8	−0.07	7.04	3.28	4.39	1.34	0.58	−0.88
Ethyl hexanoate	1,244	Fruity	MS, RI	400,137.9 ± 9,897.7	3.29	2.72	4.35	1.71	3.25	3.84	3.14
Butyl 3‐methylbutanoate	1,270	Banana, cheese	MS, RI	865.0 ± 43.5	5.68	3.49	8.46	6.04	4.93	9.81	1.21
Propyl hexanoate	1,323	Fruity	MS, RI	5,449.2 ± 206.7	−0.38	0.22	4.54	8.01	2.57	7.25	3.81
Ethyl heptanoate	1,339	Pineapple	MS, RI	24,404.1 ± 911.6	5.72	2.10	9.14	8.67	2.28	6.85	−5.08
Ethyl lactate	1,349	Fruity	MS, RI	4,402.7 ± 183.7	15.84	20.22	33.41	34.05	−0.45	31.83	18.89
Butyl hexanoate	1,418	Grape	MS, RI	15,631.4 ± 88.9	14.27	−1.37	11.28	7.00	1.61	9.64	−3.89
Ethyl octanoate	1,440	Fruity, fatty	MS, RI	35,694.7 ± 53.8	7.28	0.37	10.71	16.38	1.90	9.09	−1.20
Isopentyl hexanoate	1,465	Green	MS, RI	6,959.4 ± 101.5	8.43	−2.25	15.22	10.44	−0.01	10.72	−9.14
Pentyl hexanoate	1517	Fruity, pineapple	MS, RI	4,302.0 ± 154.9	4.01	−1.08	12.52	17.40	−1.01	11.92	1.71
Ethyl nonanoate	1542	Fruity, rose	MS, RI	678.3 ± 25.1	8.26	−2.52	15.88	25.74	2.05	15.18	5.59
Hexyl hexanoate	1615	Fruity	MS, RI	18,988.6 ± 1,050.3	4.23	−6.70	8.10	4.92	−3.85	13.47	−6.38
Ethyl phenylacetate	1803	Fruity, sweet	MS, RI	188.5 ± 9.4	0.28	−7.93	6.57	37.63	4.00	22.66	10.48
Ethyl phenylpropiolate	1910	Fruity, sweet	MS, RI	255.3 ± 12.7	3.17	2.03	8.57	29.91	9.91	24.77	13.38
Ethyl hexadecanoate	2,263	Fruity	MS, RI	289.4 ± 3.8	0.04	5.41	11.36	28.60	13.22	22.95	17.85
1‐Propanol	1,047	Alcoholic	MS, RI	1848.4 ± 54	11.05	10.71	14.46	17.56	18.45	−4.20	36.44
2‐Methyl−1‐propanol	1,106	Malty	MS, RI	569.8 ± 40.4	8.78	9.68	20.83	41.65	22.27	2.94	16.09
1‐Butanol	1,148	Malty	MS, RI	3,996.1 ± 174	6.68	4.75	8.40	15.44	4.07	9.67	6.02
3‐Methyl−1‐butanol	1,214	Malty	MS, RI	2083.5 ± 100.5	6.27	5.34	13.68	21.01	12.57	15.35	3.19
1‐Pentanol	1,254	Balsamic	MS, RI	321.6 ± 9.8	0.50	9.17	12.23	31.55	2.19	14.20	2.56
1‐Hexanol	1,356	Flower, fruity	MS, RI	2,544.1 ± 39.6	13.72	19.56	29.80	31.04	15.46	34.58	9.56
Acetic acid	1,476	Acidic, vinegar	MS, RI	1538.0 ± 40.8	26.46	21.24	12.69	−4.17	−18.65	19.43	−5.12
Butanoic acid	1648	Sweaty, rancid	MS, RI	2,633.0 ± 28.7	−0.85	2.22	−7.83	−23.12	−12.86	24.79	5.97
Pentanoic acid	1774	Sweaty	MS, RI	270.0 ± 23.8	−17.52	11.41	−16.59	−31.69	−25.85	7.06	−9.78
Hexanoic acid	1865	Sweaty	MS, RI	3,589.3 ± 194.4	−4.54	−2.93	−20.00	−42.44	−28.27	−6.73	−21.47
2‐Nonanone	1,394	soy sauce	MS, RI	242.5 ± 13.5	0.50	−0.98	17.26	16.27	−3.62	24.90	9.30
Furfural	1,480	Sweet, almond	MS, RI	552.7 ± 21.4	3.00	1.62	4.63	5.92	−1.59	1.95	−6.12
Benzaldehyde	1548	Fruity, berry	MS, RI	784.0 ± 16.5	11.40	8.06	12.96	6.73	9.50	12.48	2.14

Note

Odor descriptors of aroma compounds were from the reports by Zhang et al. (2014), and Huang et al. (2018).

Identification was based on comparing determined MS and RIs with those of reference compounds.

The concentration of each metal ion is 2 mg/L in SFB sample.

Overall, each metal had a different effect on the evolution of volatile aroma compounds. These metal cations generally promoted hydrolysis of esters, and the increase in acids helped strengthen the hydrogen bond between water and ethanol (Nose, Hojo, Suzuki, & Ueda, 2004) and reduce the organoleptic stimulation. Moreover, the decrease in the headspace concentrations of some aroma compounds implied that the hydroxyl of alcohols and the carboxyl of acids, and other aroma compounds probably bound with metal cations.

**Figure 1:**
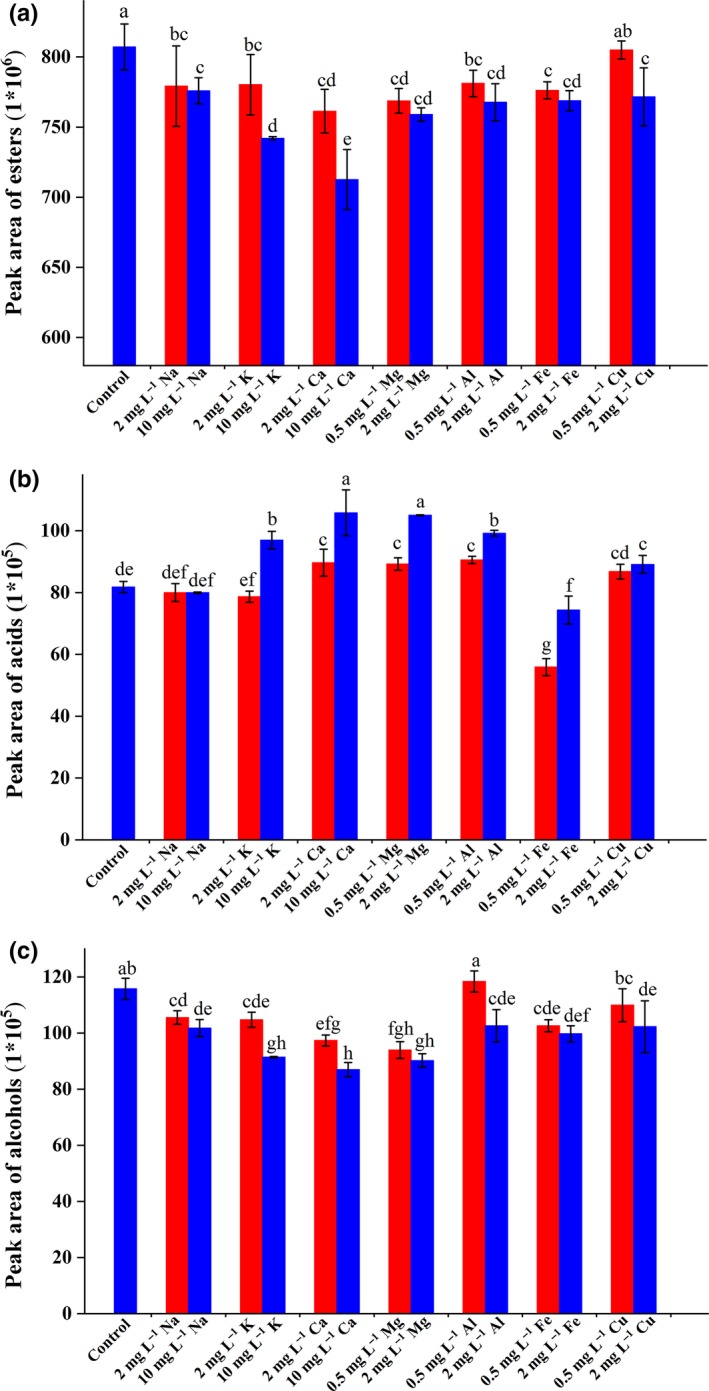
Effect of metal ions at different concentrations on esters (a), acids (b), and alcohols (c) in SFB samples. Values with different superscript letters are significantly different (*p* < .05)

Figure 1 shows the effects of adding different concentrations of these metals on the evolution of volatile aroma compounds in SFB samples. Total headspace concentrations of esters and alcohols decreased with increased metal ion concentrations (Figure 1a,c), whereas acid concentrations increased, except for the addition of Fe^3+^ (Figure 1b). This result implied that Fe^3+^ had a stronger ability to interact with carboxyl of acids in SFB than other metal ions (Na^+^, K^+^, Ca^2+^, Mg^2+^, Al^3+^, and Cu^2+^). Esters and acids were the main aroma compounds in baijiu. Total acid content increased and total ester content decreased during the aging period (Qiao, Zhang, & Wang, 2013). Therefore, the above results indicated that the quality of aged SFB was associated with Na, K, Ca, Mg, Al, Fe, and Cu, and these metals had an aging‐advancing function.

### 3.3 Effects of main metals on fluorescence of SFB and acids solution

**Figure 2:**
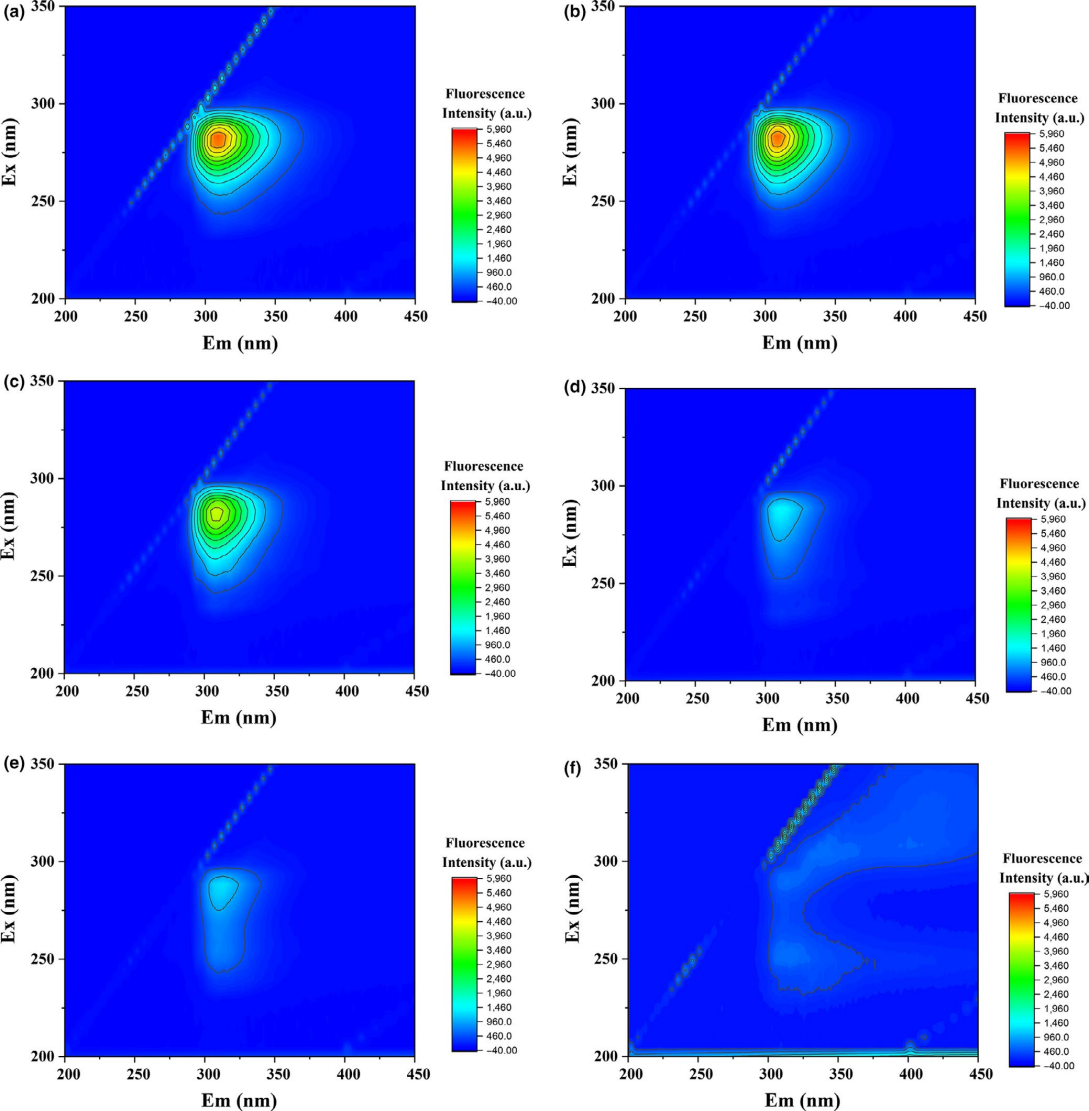
Fluorescence EEM landscapes of SFB samples. (a), (b), (c), (d), (e), and (f) are SFB samples stored in pottery jar for 0, 1, 2, 3, 5, and 10 years, respectively

Fluorescence analysis was used to study the mechanism of interaction between metals (Na, K, Ca, Mg, Al, Fe, and Cu) and SFB. Fluorescence EEM landscapes of fresh and stored SFB samples are shown in Figure 2, and their spectral characteristics are listed in Table 3. Maximum fluorescence intensity decreased with increasing aging time in pottery jar. Maximum excitation and emission wavelengths moved from 280 nm to 250 nm and from 309 nm to 316 nm, respectively. Fluorescence of components, such as flavonoids, salicylic acid, and (–)‐epigallocatechin gallate with β‐lactoglobulin, was changed after binding with Cu^2+^, Sn^2+^, Zn^2+^, Mn^2+^, Al^3+^, and Fe^3+^ through fluorescence analysis (Lavrik & Mulloev, 2010; Zhang, Sahu, Xu, Wang, & Hu, 2017; Zhang, Shi, Sun, Xiong, & Peng, 2011). Hence, the above‐mentioned change in SFB fluorescence might be ascribed to the binding of some metals released from pottery jar to the components in SFB during storage.

**Table 3:** Fluorescence EEM spectral characteristics of SFB samples aged in pottery jar

SFB samples	Peak position *λ*_ex_/*λ*_em_ (nm/nm)	Intensity (a.u.)
P0	280/309	5,371
P1	280/308	5,355
P2	280/307	4,197
P3	285/311	1,441
P5	285/312	1,286
P10	250/316	145

Note

P0, P1, P3, P5, and P10 are SFB samples stored in pottery jar for 0, 1, 3, 5, and 10 years, respectively.

**Figure 3:**
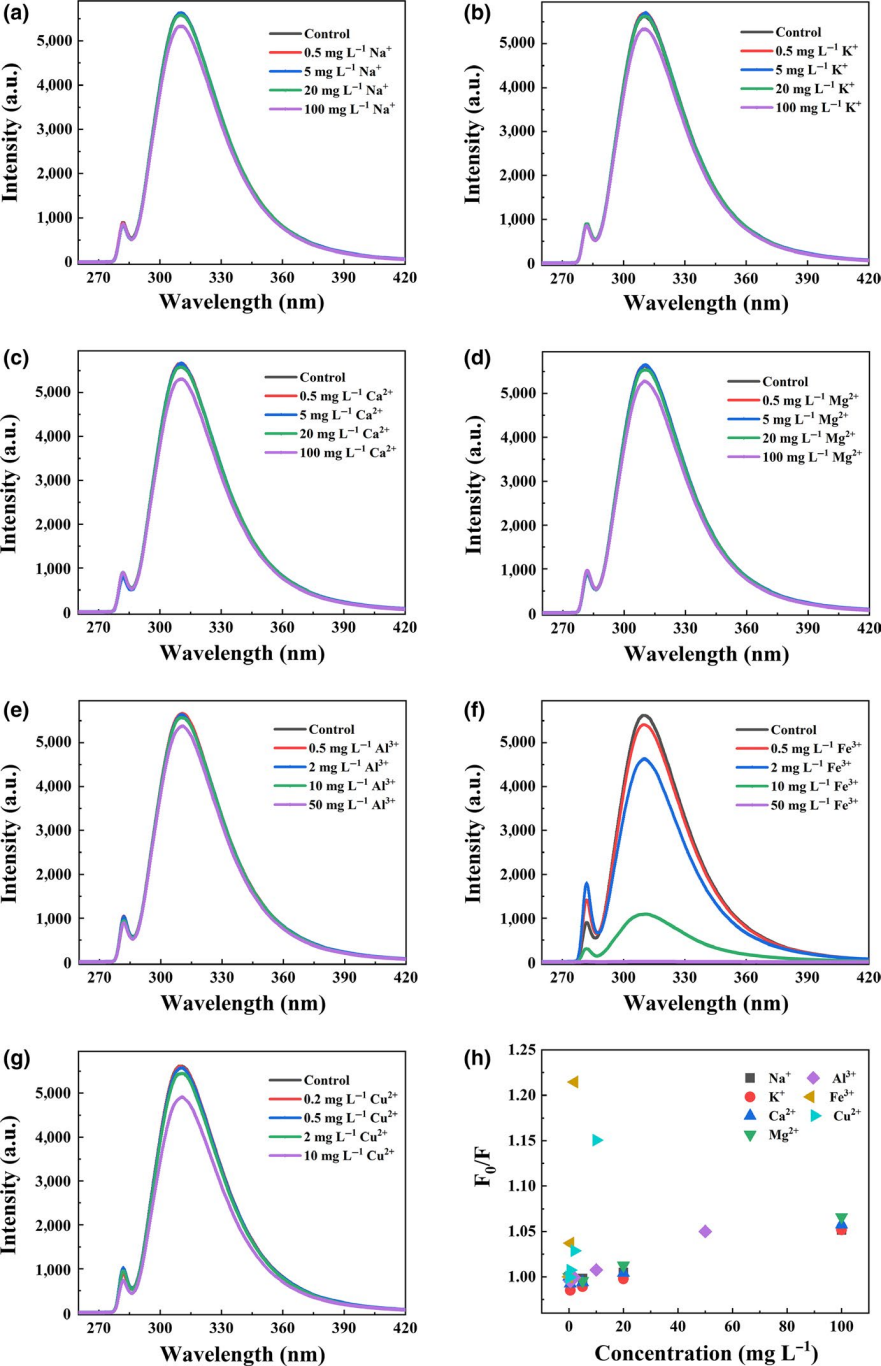
Effect of metal ions on SFB fluorescence intensity at different concentrations (a‐g). The plots for SFB fluorescence quenching by metal ions (h). *λ*_ex_ = 280nm

Fluorescence quenching is a useful technique to determine binding affinities of metals to target components (De Costa & Jayasinghe, 2004; Tan, Kim, & Kool, 2011). Different concentrations of Na^+^, K^+^, Ca^2+^, Mg^2+^, Al^3+^, Fe^3+^, and Cu^2+^ were separately added into fresh SFB and acid solutions. Changes in SFB fluorescence intensity in the presence of Na^+^, K^+^, Ca^2+^, Mg^2+^, Al^3+^, Fe^3+^, and Cu^2+^ are shown in Figure 3. Fluorescence intensity of SFB was slightly changed by adding Na^+^, K^+^, Ca^2+^, Mg^2+^, and Al^3+^ at a low concentration (Figure 3a–e) but gradually decreased with increased concentrations of Fe^3+^ and Cu^2+^ (Figure 3f,g). Plots of F_0_/F for SFB versus metal ions at different concentrations exhibited that the binding affinity of Fe^3+^ was higher than that of Cu^2+^ (Figure 3h). Fluorescence intensities of acid solutions were almost unchanged after adding different concentrations of Na^+^, K^+^, Ca^2+^, Mg^2+^, and Al^3+^ into the acids solution but decreased with increased concentrations of Fe^3+^ and Cu^2+^ (Figure S2). These fluorescence quenching data showed that Fe^3+^ and Cu^2+^ had higher binding affinity with carboxyl of acetic acid, butanoic acid, and hexanoic acid in 70% (v/v) ethanol/water solution than Na^+^, K^+^, Ca^2+^, Mg^2+^, and Al^3+^.

Irto et al. (2018) reported that Fe^3+^, Al^3+^, Cu^2+^, and Ca^2+^ could bind with a compound derived from nitrilotriacetic acid. Hussain, Rahim, and Farooqui (2012) showed that the binary complexes of mandelic acid ligand with Fe^3+^ and Cu^2+^ had higher stability than those with Cd^2+^, Zn^2+^, Ni^2+^, and Co^2+^. Their results indicated that these metals, especially Fe^3+^ and Cu^2+^, could chelate or bind with different organic compounds, which is consistent with our fluorescence analyses. Besides, Fe^3+^ and Cu^2+^ even have oxidative potential and may oxidize aroma compounds in SFB (Benítez, Castro, Antonio Sanchez, Pazo, & Barroso, 2002; Danilewicz, 2007; Iwegbue, Overah, Bassey, & Martincigh, 2014). Hence, we inferred that some metal ions could bind with or oxidize aroma compounds, especially acids in SFB, and thereby form colloidal particles or complexes during the storage. This study would be helpful to understand why metals can reduce organoleptic stimulation of alcoholic beverages.

## 4 CONCLUSION

Some metals were dissolved out from pottery jar and stainless‐steel vessel into the SFB during storage. Their concentrations were lower than the recommended allowable daily intake. When SFB was stored in pottery jar, the concentrations of Na, K, Ca, Mg, Al, and Fe remarkably increased, and concentrations of Ba, Be, Cu, Li, Mn, Ni, Sr, Ti, and Zn slightly increased. When SFB was stored in stainless‐steel vessel, the concentrations of Fe and Cu greatly increased, and concentrations of Cr, Mn, Ni, Sr, Ti, and Zn slightly increased. Metals released from pottery jar and stainless‐steel vessel into SFB were consistent with the main components of the two containers. It is worth noting that the Cu released in SFB should be the impurity, which was introduced from welding process in production of the stainless‐steel vessel. Headspace concentrations of most esters, alcohols, ketone, furan, and aldehyde in fresh SFB decreased, whereas headspace concentrations of most acids increased after adding Na^+^, K^+^, Ca^2+^, Mg^2+^, Al^3+^, Fe^3+^, and Cu^2+^. These metal cations promoted the decrease in total ester content and the increase in total acid content, indicating that these metal cations had an aging‐advancing function. Furthermore, maximum fluorescence intensity of SFB decreased with increasing aging time. This result might be ascribed to the binding between metals released from the container and aroma components, especially acids in SFB during storage. This binding can promote the formation of metal‐aroma compound complexes or colloidal particles. This finding is helpful to explain the release of metal cations and the formation of aroma compounds of baijiu during storage, which is significant for modulating the flavor and sensory properties of baijiu.

## CONFLICT OF INTEREST

The authors declare that they do not have any conflict of interest.

## ETHICAL APPROVAL

This study does not involve any human or animal testing.
