# Supplementary material for: Effects of metals released in strong‐flavor baijiu on the evolution of aroma compounds during storage
Source: Food Sci Nutr. 2020 Feb 20;8(4):1904–13. doi: 10.1002/fsn3.1475 (PMC7174237; doi:10.1002/fsn3.1475)
Supplement: Supplementary file 1 [file FSN3-8-1904-s001.docx]

**Supporting Information**

**Effects of metals released in strong-flavour baijiu on the evolution of aroma compounds during storage**

Zhangjun Huang,^1^ Yunhang Zeng,^1*^ Wenhu Liu,^2, 3^ Songtao Wang,^2,3^ Caihong Shen,^2,3^ Bi Shi^1^

1 College of Biomass Science and Engineering, Sichuan University, Chengdu, 610065, China

2 National Engineering Research Center of Solid-State Brewing, Luzhou, 646000, China

3 Luzhou Laojiao Co.,Ltd., Luzhou, 646000, China

*Correspondent: E-mail: zengyunhang@scu.edu.cn


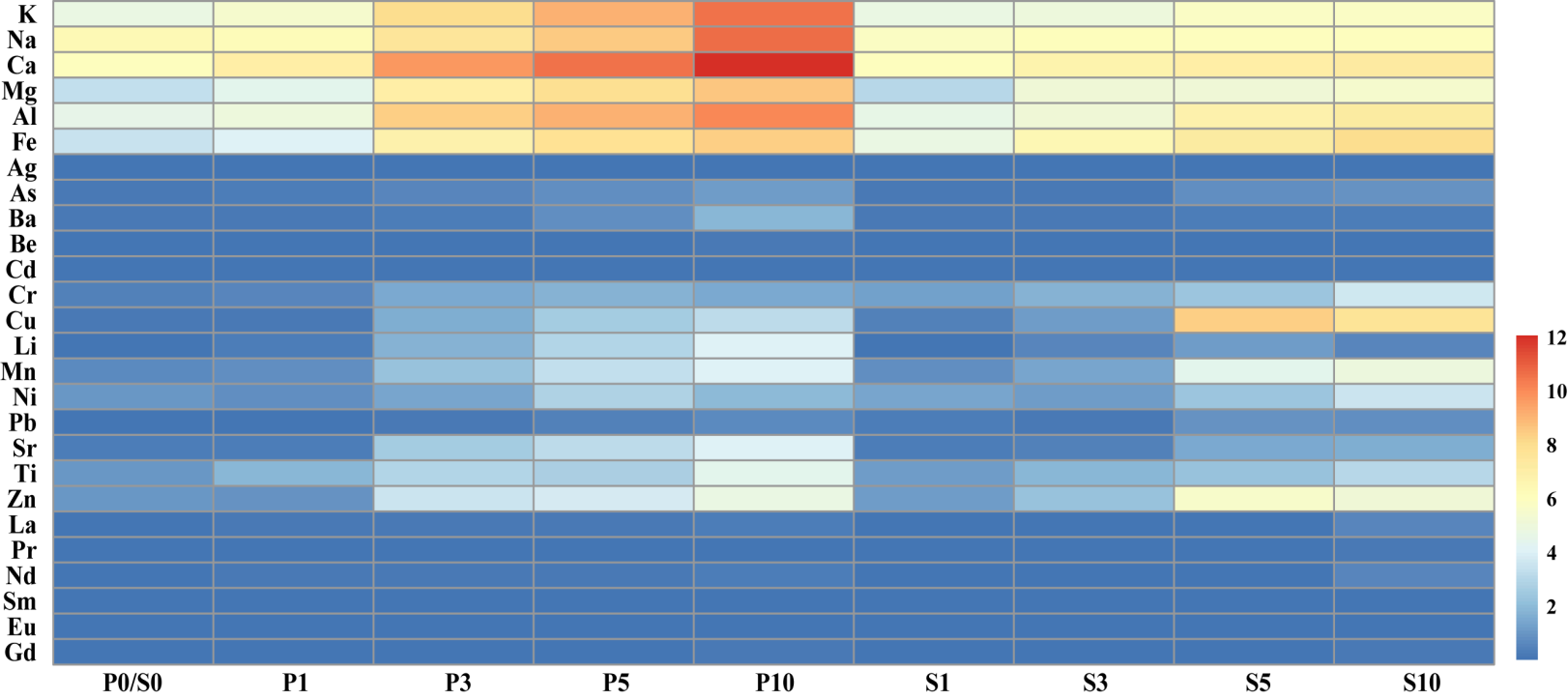


**Figure S1** Heat map visualization of metals in SFB samples stored in pottery jar and stainless-steel vessel. The relative concentrations of the metals are depicted by color intensity. Red, yellow and blue represent the high, middle and low concentrations.


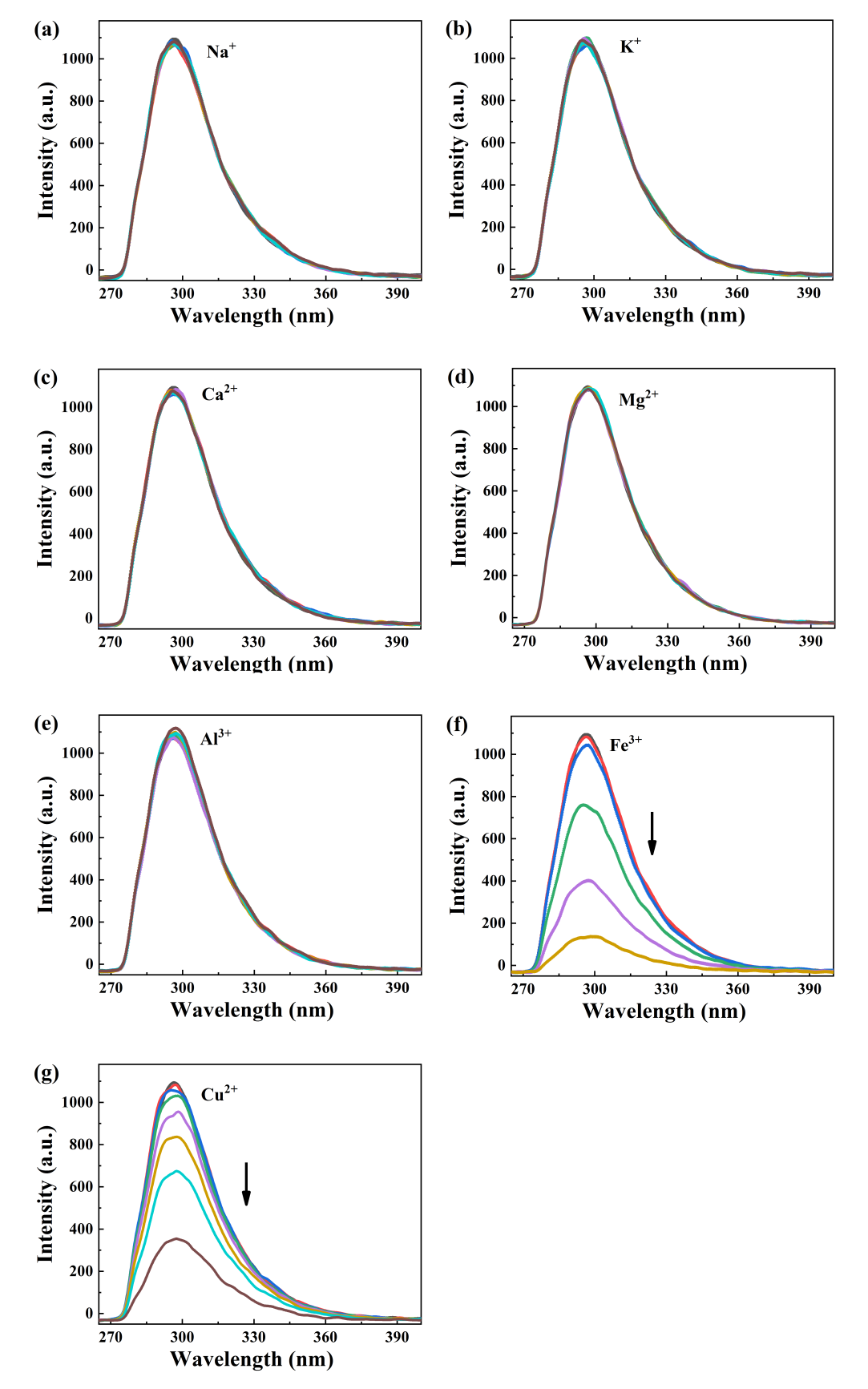


**Figure S2** Quenching effects of metal ions on fluorescence intensity of acids (a-g). λ_ex_=220nm. Figures (a)-(e) and (g), 0, 0.1, 0.5, 2, 5, 10, 20 and 50 mg L^-1^ of Na^+^, K^+^, Ca^2+^, Mg^2+^, Al^3+^ and Cu^2+^. Figure (f), 0, 0.1, 0.5, 2, 5 and 10 mg/L of Fe^3+^.
